# Bioinspired Control Architecture for Adaptive and Resilient Navigation of Unmanned Underwater Vehicle in Monitoring Missions of Submerged Aquatic Vegetation Meadows

**DOI:** 10.3390/biomimetics9060329

**Published:** 2024-05-30

**Authors:** Francisco García-Córdova, Antonio Guerrero-González, Fernando Hidalgo-Castelo

**Affiliations:** Department of Automation, Electrical Engineering, and Electronic Technology, Polytechnic University of Cartagena, 30203 Cartagena, Spain; fgc_master@upct.es (F.G.-C.); fernando.hidalgo2@edu.upct.es (F.H.-C.)

**Keywords:** UUV, underwater robots, bioinspired control, artificial neural networks, self-organizing networks trajectory tracking, fault-tolerant control, submerged aquatic vegetation

## Abstract

Submerged aquatic vegetation plays a fundamental role as a habitat for the biodiversity of marine species. To carry out the research and monitoring of submerged aquatic vegetation more efficiently and accurately, it is important to use advanced technologies such as underwater robots. However, when conducting underwater missions to capture photographs and videos near submerged aquatic vegetation meadows, algae can become entangled in the propellers and cause vehicle failure. In this context, a neurobiologically inspired control architecture is proposed for the control of unmanned underwater vehicles with redundant thrusters. The proposed control architecture learns to control the underwater robot in a non-stationary environment and combines the associative learning method and vector associative map learning to generate transformations between the spatial and velocity coordinates in the robot actuator. The experimental results obtained show that the proposed control architecture exhibits notable resilience capabilities while maintaining its operation in the face of thruster failures. In the discussion of the results obtained, the importance of the proposed control architecture is highlighted in the context of the monitoring and conservation of underwater vegetation meadows. Its resilience, robustness, and adaptability capabilities make it an effective tool to face challenges and meet mission objectives in such critical environments.

## 1. Introduction

Submerged aquatic vegetation (SAV) is vital for maintaining marine biodiversity, preventing erosion, and regulating water quality. SAV habitats serve as nurseries, feeding grounds, and refuges for various marine species, contributing to the overall health of marine ecosystems [1]. However, SAV is facing significant threats from human activities, such as pollution, climate change, and habitat alteration, leading to its rapid decline.

The Mar Menor, a large saline coastal lagoon in Spain, is currently experiencing issues related to water quality and the loss of submerged aquatic vegetation, despite its unique biodiversity and importance as a habitat for numerous marine species [2]. The proliferation of the invasive algae Caulerpa Prolifera, which now covers a significant portion of the Mar Menor’s seabed, is a major concern due to its potential environmental impacts. The continuous monitoring of Caulerpa Prolifera’s distribution, density, and expansion is crucial for assessing its effects on local biodiversity, water quality, and aquatic ecosystems, as well as identifying areas where its growth is most aggressive, allowing for the timely implementation of preventive or corrective measures [3]. Advanced technologies, such as remote sensing systems, sonars, echo sounders, underwater sensors, and underwater robots, are essential for the accurate and detailed monitoring of underwater aquatic vegetation in aquatic ecosystems [4,5]. Underwater robots, including remotely operated vehicles (ROVs), autonomous underwater vehicles (AUVs), and unmanned underwater vehicles (UUVs), are particularly effective tools for monitoring submerged aquatic vegetation, as they can collect precise data on its distribution, density, and condition [6,7,8]. These robots generate high-resolution images and geospatial data, enabling the creation of three-dimensional maps of underwater ecosystems, which provide valuable information on the distribution, structure, and relationship of aquatic vegetation with other habitat elements, as well as aiding in assessing ecosystem health and monitoring invasive species.

However, when operating underwater robots at close range to the seabed to capture images and videos, there is a risk of algae and other aquatic plants becoming entangled in the propellers, which can cause damage and affect the operation of the vehicle. This situation highlights the need for fault-tolerant control (FTC) strategies to ensure the resilience and reliability of underwater robots in challenging environments.

Fault-tolerant control is a critical aspect of underwater robotics, as it enables the system to maintain its stability and performance in the presence of faults or failures, such as propeller entanglement. FTC techniques aim to detect, isolate, and accommodate faults in real time, ensuring that the robot can continue its mission despite the occurrence of unexpected events [9]. By incorporating fault-tolerant control strategies, underwater robots can adapt to adverse situations and maintain optimal control and stability.

Bioinspired systems emerge as promising approaches for the development of resilient and adaptive control algorithms, including fault-tolerant control strategies. Biomimicry is based on the idea that nature has evolved over millions of years to find the optimal solutions to various challenges, and that we can learn from these designs and strategies to apply them in the fields of engineering and technology. Bioinspired systems, on the other hand, are those that are inspired by the principles, structures, and functions of biological systems to create innovative and efficient solutions [10].

Bioinspired control algorithms [11] are a powerful tool that exemplifies resilience and the ability to reconfigure in situations of a sudden loss of capabilities, such as in the case of a locked propeller. These algorithms are inspired by biological systems and behaviors observed in nature to adapt and respond effectively to adverse situations. These bioinspired algorithms mimic the principles of self-organization, adaptability, and cooperation present in living organisms. They can adjust control parameters, redistribute tasks among functional components, and adapt motion strategies to compensate for the defective part. For example, in the case of a blocked propeller, a bioinspired algorithm could detect the failure and automatically reconfigure the distribution of power between the remaining propellers, ensuring that the robot can continue moving and fulfill its mission despite the failure.

Bioinspired algorithms and artificial neural networks, such as self-organizing neural maps, also known as Kohonen maps or SOMs (self-organizing maps) [12,13], are powerful tools used in route planning for autonomous underwater robots. These maps are based on the structure and functioning of the human brain and are used to organize and represent complex information in two-dimensional or three-dimensional space.

In the specific case of learning to cope with a stuck propeller situation, self-organizing neural maps can play a crucial role in the fault-tolerant trajectory planning of autonomous robots. These maps allow the robot to learn and adapt to the presence of a blocked propeller and find alternative solutions to maintain its operation and meet its objectives. The neural map training process involves presenting input data that describe the environment and movement constraints of the robot when it encounters a locked propeller. The neural map learns to recognize patterns associated with a locked propeller and maps them to a specific region of the map. As the robot interacts with different locked propeller scenarios, the neural map updates and refines its representation to adapt to new situations. This allows the robot to learn incrementally and improve its ability to plan optimal trajectories in the presence of locked propellers. The application of self-organizing neural maps in the case of a blocked propeller provides the robot with an efficient mechanism to learn and adapt to faulty or malfunctioning situations. By recognizing and mapping specific patterns in the environment, the robot can make informed decisions and find alternative solutions that allow it to continue operating effectively and fulfill its assigned tasks.

This work presents an application case of a bioinspired control architecture applied to a UUV with propulsion redundancy, which allows for reconfiguring the propulsion patterns to overcome the problem of propeller loss and continue with the mission objectives. This bioinspired control architecture combines the principles of biomimicry and bioinspired systems with advanced artificial intelligence and machine learning techniques, such as self-organizing neural maps, to achieve resilient and adaptive control in failure situations, including fault-tolerant control strategies.

The results obtained in this work demonstrate the effectiveness of bioinspired and biomimetic approaches in the development of robust and resilient control systems for UUVs, with a particular focus on fault-tolerant control. The ability of these systems to learn, adapt, and reconfigure in real time in the face of failures and disturbances makes them valuable tools for the monitoring and conservation of marine ecosystems, especially in challenging environments such as submerged aquatic vegetation habitats.

The integration of bioinspired and biomimetic concepts and techniques, along with fault-tolerant control strategies, in the design of control algorithms for underwater robots opens up new possibilities for the development of more robust, adaptable, and efficient systems. These approaches allow us to learn from nature’s wisdom and apply its principles to address current technological challenges, such as monitoring and protecting vulnerable marine ecosystems.

The rest of this document is organized as follows. Section 2 reviews the work on bioinspired control techniques and fault-tolerant control strategies upon which this work is based. Section 3 presents the proposed materials and methods, describing the UUV, the bioinspired algorithms implemented for control, and the operating procedures. Section 4 shows the results obtained in the test and validation experiments. Section 5 discusses the results obtained, and Section 6 presents the conclusions of this work.

## 2. Related Works

Robotic resilience is a rapidly growing area of research that seeks to develop robotic systems capable of adapting and recovering from failures, disturbances, and changes in the environment. In this context, bioinspired and biomimetic algorithms have emerged as promising approaches to endow robots with resilience capabilities similar to those observed in biological systems [14]. These algorithms are based on the emulation of the principles and mechanisms of adaptation, learning, and robustness present in nature, with the aim of creating more resilient and autonomous robotic systems [15]. Fault-tolerant control (FTC) is a critical aspect of robotic resilience, as it enables the system to maintain its stability and performance in the presence of faults or failures [16]. FTC techniques aim to detect, isolate, and accommodate faults in real time, ensuring that the robot can continue its mission despite the occurrence of unexpected events [17].

Biological organisms have evolved over millions of years to develop resilience strategies that allow them to cope with dynamic, uncertain, and challenging environments. These capabilities are based on mechanisms such as redundancy, modularity, self-organization, and learning, which allow organisms to maintain their functionality and performance even under adverse conditions [18]. Bioinspired and biomimetic approaches to fault-tolerant control draw inspiration from these biological mechanisms, seeking to develop robotic systems that can adapt and recover from faults in a manner similar to living organisms [19].

Another bioinspired approach to robotic resilience is based on reinforcement learning, inspired by the learning and adaptation mechanisms observed in animals [20]. In this approach, robots learn through interaction with the environment, receiving rewards or punishments based on the consequences of their actions. This learning process allows robots to develop robust and adaptive behavioral strategies capable of coping with changes and perturbations in the environment [21]. Reinforcement learning has been applied to fault-tolerant control, enabling robots to learn optimal control policies in the presence of faults or failures [22]. By exploring the space of possible actions and receiving feedback on their performance, robots can learn to adapt their behavior to compensate for faults and maintain a stable operation.

In addition to the mentioned approaches, there are other bioinspired and biomimetic algorithms that have been applied to improve robotic resilience. Algorithms based on synaptic plasticity and Hebbian learning, inspired by the learning mechanisms of the brain, have been used to provide robots with adaptation and recovery capabilities in the face of changes in the environment or their own dynamics [23]. These approaches have been extended to fault-tolerant control, enabling robots to adapt their control strategies in response to faults or failures [24]. By adjusting the weights of neural connections based on the correlation between input and output signals, these algorithms can learn to compensate for faults and maintain a stable performance.

The neural models that inspire this work are based on the way in which neurons in the primary motor areas (MI) of the central nervous systems (CNSs) of animals are activated when they carry out a specific task. These models simulate learning based on how biological organisms respond to various stimuli and adapt to their environment, analogous to how this occurs in nature [25,26]. Recently, various models of adaptive control systems that mimic the function of the cerebellum have been developed [27,28]. These models are used to create movement trajectories using unsupervised Hebbian learning, an approach that is inspired by the synaptic plasticity mechanisms observed in the brain [29,30]. These mechanisms allow for the connections between neurons to be strengthened or weakened depending on neuronal activity, enabling learning and adaptation. In the context of fault-tolerant control, cerebellar-inspired models have been proposed to enable robots to adapt their motor commands in response to changes in their dynamics or the environment [31]. By learning to predict the sensory consequences of their actions and comparing them with the actual sensory feedback, these models can detect and compensate for discrepancies caused by faults or failures.

Various neural control architectures have incorporated self-organizing maps (SOMs), a mathematical mechanism that enables the learning of the sensorimotor mapping necessary to model both direct and inverse models in the field of robotic control [24,32]. These architectures, inspired by biological systems, are based on the principles of unsupervised learning and self-organization observed in the brain, which gives them the ability to adapt and generalize their performance in the face of novel situations. SOMs have been applied to fault-tolerant control, enabling robots to learn and adapt their control strategies in response to faults or failures [33]. By organizing the sensory and motor spaces into topologically preserved maps, SOMs can detect and isolate faults, and reconfigure the control system to maintain a stable performance.

In [34], a self-organized neuronal model called Direction-to-Rotation Effector Control Transform (DIRECT) was proposed, designed to achieve motor equivalence. This biomimetic model performs a coordinate transformation, converting spatial directions into actuator velocities. Learning in this model is based on a vector associative map (VAM), which gains the ability to transform multiple combinations of visual or spatial positions into a single invariant target position in three-dimensional space [35]. Likewise, a real-time neural network model known as the vector integration to endpoint (VITE) model was developed. This model was used to simulate quantitative neural and behavioral data related to planned and passive arm movements [36]. Both the DIRECT and VITE models are inspired by biological systems and manage to capture the fundamental characteristics of motor control observed in nature. These bioinspired principles are then applied to the control of robotic systems, allowing for the development of more efficient and adaptive algorithms and control strategies which emulate the capabilities of biological systems to generate smooth, coordinated, and precise movements. In the context of fault-tolerant control, the DIRECT and VITE models have been extended to enable robots to adapt their motor commands in response to changes in their dynamics or the environment caused by faults or failures [37]. By learning to transform spatial goals into appropriate motor commands, these models can provide robotic systems with the ability to maintain stable and precise movements even in the presence of faults.

The control algorithms for UUVs proposed in this paper are based on the aforementioned bioinspired neural control models. These algorithms were initially developed in the field of mobile robotics and have since been successfully adapted and applied to autonomous marine robotics. Several significant works have demonstrated the effectiveness of these bioinspired approaches in the context of mobile robotics and the control and navigation of autonomous marine vehicles.

In mobile robotics, reinforcement learning has been widely explored as an approach to develop adaptive and autonomous behaviors. A particular form of reinforcement learning, known as operant conditioning, has been investigated in the context of applying biological learning theories to avoidance and approach behaviors in mobile robots. Operant conditioning is based on the idea that behaviors followed by positive consequences (rewards) tend to be strengthened and repeated, while those followed by negative consequences (punishments) tend to be weakened and avoided. In [38], a neural network for obstacle avoidance based on a model of classical and operant conditioning was presented. This network uses reinforcement signals to adjust synaptic weights and adaptively shape the robot’s behavior. This research was built on previous work exploring the application of unsupervised neural networks for the low-level control of wheeled mobile robots [39,40]. In these studies, neural networks were developed to generate adaptive behaviors in non-stationary environments, demonstrating both noise resistance and stability in hardware implementations.

Furthering this line of research, in [41], a neural network that learned to control approach and avoidance behaviors in a mobile robot using operant conditioning was presented. Learning, which required no supervision, took place as the robot moved around an environment cluttered with obstacles and light sources. The neural network required no knowledge of the geometry of the robot or of the quality, number, or configuration of the robot’s sensors. In this work, a detailed presentation of the model and the results with the Khepera and Pioneer 2 mobile robots was provided.

Due to their fault-tolerant navigation and reconfiguration capabilities, these neuro-controllers have been employed in vehicles designed for long-term applications. In [42], an autonomous vehicle for long-term observation and monitoring was proposed, integrating photovoltaic panels and a methanol fuel cell for energy autonomy, along with a neurobiologically inspired control architecture for intelligent navigation. This architecture, originally designed for nonholonomic mobile robots, included a kinematic adaptive neuro-controller for trajectory tracking and an adaptive neuro-controller for obstacle avoidance, enabling autonomous navigation and adaptation to environmental changes. The bioinspired control algorithms enhanced resilience and adaptability, showcasing the potential for sustainable and intelligent robotics in real-world scenarios.

Subsequently, in [43], these bioinspired control algorithms were applied in the field of marine robotics. They were used in a marine robotic system consisting of an autonomous surface vessel and UUVs designed for the long-term monitoring of oil spills. Resilient capabilities and fault tolerance were required due to the harsh operating conditions encountered in oil spill environments. The system’s resilience was provided by bioinspired algorithms implemented in a modular software architecture and controlled by redundant devices. This ensured the necessary robustness for operation under the challenging conditions typically found in long-term oil spill operations.

In summary, fault-tolerant control is a critical aspect of robotic resilience, enabling robotic systems to maintain their stability and performance in the presence of faults or failures. Bioinspired and biomimetic approaches to fault-tolerant control draw inspiration from the principles and mechanisms of adaptation, learning, and robustness present in biological systems, seeking to develop robotic systems that can adapt and recover from faults in a manner similar to living organisms. Reinforcement learning, synaptic plasticity, and Hebbian learning are some of the bioinspired approaches that have been applied to fault-tolerant control, providing robotic systems with the ability to learn and adapt their control strategies in response to faults or failures. Neural control architectures, such as self-organizing maps and hierarchical controllers, have also been applied to fault-tolerant control, enabling robots to detect and compensate for faults at different levels of the control system. The DIRECT and VITE models, inspired by biological motor control, have been extended to enable robots to adapt their motor commands in response to changes in their dynamics or the environment caused by faults or failures. Additionally, the application of operant conditioning and unsupervised neural networks for the low-level control of mobile robots has been explored, demonstrating their effectiveness in generating adaptive and robust behaviors in non-stationary environments. These bioinspired control algorithms have been further applied to autonomous vehicles for long-term observation and monitoring tasks, such as solar-powered robots and marine robotic systems for oil spill monitoring, showcasing their potential for enhancing robotic resilience in real-world scenarios.

As robotic systems become increasingly complex and are deployed in more challenging environments, the development of effective fault-tolerant control strategies becomes crucial. Bioinspired and biomimetic approaches offer a promising direction for designing robotic systems that can adapt and recover from faults, drawing inspiration from the remarkable resilience and adaptability of biological systems.

## 3. Materials and Methods

### 3.1. Experimental Vehicle

**Figure 1:**
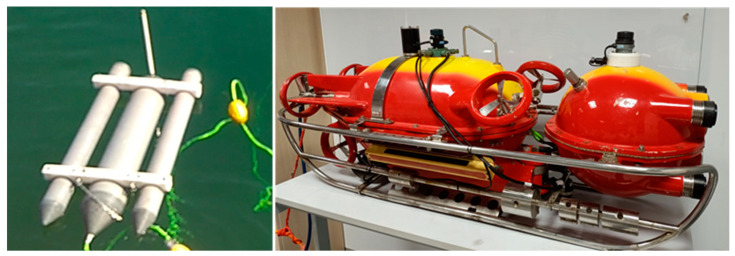
The prototype UUV.

The prototype UUV system is composed of an underwater vehicle and a buoy. The buoy is towed by the underwater vehicle and is connected via an umbilical cable, as depicted in Figure 1. The entire system operates autonomously from the control base, with communications being facilitated through wireless technology.

The underwater vehicle represents a modification of an existing commercial model and is composed of two hulls. One hull accommodates five motors for propulsion and maneuvering, along with a battery pack and electronics. The second hull, spherical in shape, constitutes the vehicle’s “head” and houses the sensors. This vehicle is designed to operate at a depth of up to 300 m.

Regarding velocity, the UUV is capable of reaching a maximum horizontal speed of 4 knots and can maintain its position against horizontal currents of up to 3 knots. The vehicle’s agility across all axes is facilitated by a combination of thrusters: two horizontal thrusters enable forward and backward movements, two vertical thrusters allow for ascending and descending, and a transversal stern thruster, referred to as the rudder thruster, provides the capability for right and left turns.

In terms of its physical specifications, the UUV has a weight of 163.4 kg in air and displaces 163.8 cubic decimeters of water. The vehicle is designed with a positive buoyancy of approximately 300 g, ensuring that it will slowly rise to the surface if the controls are disengaged.

For missions aimed at monitoring underwater vegetation, the vehicle is equipped with a suite of essential equipment. This includes cameras accompanied by spotlights for visual documentation, side-scan sonar for comprehensive sonar imaging, and advanced navigation systems, such as Doppler Velocity Log (DVL) estimation and GPS. Additionally, it is equipped with depth sensors for accurate measurements of underwater depths, acoustic positioning systems for precise location tracking, and enhanced navigation capabilities facilitated by imaging sonar technology.

The UUV is specially equipped with advanced instruments designed for the exploration of underwater vegetation. These instruments comprise:

- Tritech Seaking Side-Scan Sonar: This sonar operates at a frequency of 325 kHz, featuring a vertical beamwidth of 30 degrees and a horizontal beamwidth of 1 degree. Its maximum operational range is 200 m, with a pulse length of 400 microseconds.
- Tritech Micron Echosounder DST Acoustic Altimeter: This device functions at a frequency of 500 kHz, with a conical beamwidth of 6 degrees. It provides a maximum detection range of 50 m and a minimum range of 0.5 m, featuring a digital resolution of 1 mm.
- NavQuest 600 Micro Doppler (Doppler Velocity Log, DVL) from Linquest Inc.: This compact and integrated DVL acts as a critical navigation and positioning tool for the underwater vehicle. It operates at a frequency of 600 kHz and is capable of functioning at depths of up to 800 m, ensuring a reliable and accurate performance.
- Tritech Micron DST Imaging Sonar: This sonar employs CHIRP technology centered at 700 kHz. It features a vertical beamwidth of 35 degrees and a horizontal beamwidth of 3 degrees. The sonar has a maximum detection range of 75 m and a minimum range of 0.3 m. It is capable of scanning a variable sector of up to 360 degrees.
- Optical Vision Cameras: The UUV is equipped with two high-quality optical vision cameras designed for real-time vision and photography. These cameras provide SVGA resolution and are capable of both day and night vision functionality. Utilizing a 1/4” progressive scan RGB CMOS image sensor, they feature a varifocal lens with a focal range of 3–8 mm, enabling a broad field of view. The cameras exhibit low light sensitivity, with 0.3 lux in color mode and 0.05 lux in black and white mode.
- Tritech MicronNav System: For the accurate determination of the UUV’s position, it utilizes an acoustic transponder from the Tritech MicronNav System. This transponder receives signals from an Ultra Short Baseline (USBL) type transducer stationed at the surface control station. Operating within a frequency band of 20–28 kHz, the transponder has a horizontal range of 500 m and a vertical range of 150 m, delivering a precise bearing accuracy to within ±0.2 m.

The control hardware possesses the computing capabilities required for the system to execute its control functions and to implement the bioinspired control architecture. This neuro-inspired control architecture operates solely on the system’s CPU, housed within the underwater vehicle. The neuro-controllers endow the system with the ability to withstand failures in the UUV’s propulsion and control system, enhancing its operational resilience.

The system features an Ethernet network that links the submarine and towed buoy’s equipment, including the real-time controllers and sensors (cameras and sonar) of both vehicles. The subsea vehicle employs a distributed CANopen fieldbus-based control system within the UUV. In addition to the vehicle’s computer, an sbRIO-9606 Programmable Automation Controller is integrated to facilitate the bridging of Ethernet and CAN networks. This controller serves as an information server for the system’s CPU, ensuring its reliable and deterministic operation.

The UUV’s CANopen network comprises four nodes: two located in the propulsion hull and two within the spherical front hull section of the vehicle. This network is responsible for controlling the actuator thrusters, head movement, and focus control, and for reading data from pressure, flood, humidity, Doppler Velocity Log (DVL), and other sensors.

### 3.2. Navigation System Based on Biologically Inspired Neural Algorithms

**Figure 2:**
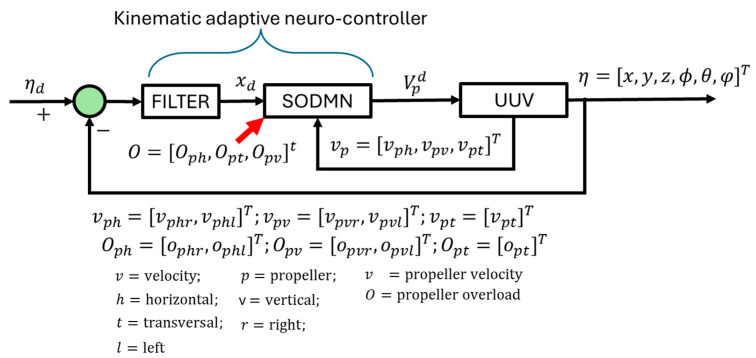
SODMN adaptive navigation of the UUV.

The primary objective of the proposed active navigation system is to perform target positioning, as well as trajectory tracking, and to adapt to adverse situations while maintaining a stable and effective performance (for example, continuing a mission with the minimum number of required actuators). The UUV is equipped with an advanced intelligent control architecture, inspired by neurobiological principles, designed specifically for trajectory tracking. This architecture is based on a Self-Organizing Direction Mapping Network (SODMN). The structure and functioning of this network are illustrated in Figure 2.

The inputs of the SODMN are the vehicle’s position, and the outputs are commands to the UUV’s thrusters/actuators (five commands to thrusters). The spatial coordinates of the UUV are $\eta ={\left[x\;y\;z\;\varphi \;\theta \;\psi \right]}^{T}$, where the orientation angles are given by $\phi ={\left[\varphi \;\theta \;\psi \right]}^{T}$. The rotational movements around three axes (longitudinal/*X*-axis, transverse/*Y*-axis, and vertical/*Z*-axis) are known as roll (φ), pitch (θ), and yaw (ψ), respectively.

For dynamic positioning in path tracking, a filter is incorporated into the control system architecture which smothers the error signal in reaching objectives. The tracking spatial error (*e*) is computed and filtered by a PID to obtain the desired spatial direction vector ($x_{d}$) and is given as

(1) $$x_{d}\left(t\right)=K_{p}e\left(t\right)+K_{i}\int edt+K_{d}\frac{de}{dt},$$

where t is the time, $K_{p}$ is the proportional gain, $K_{i}$ is the integral gain, and $K_{d}$ is the derivative gain. The gain parameters are adjusted based on simple knowledge of the spatial behavior of the UUV.

The SODMN receives the filtered direction error ($x_{d}$), as well as the vector of thruster velocities ($V_{p}$), generating spatial coordinate transformations at underwater vehicle thruster velocity commands ($V_{p}^{d}$). In a situation where the UUV receives a desired target without a defined trajectory, the SODMN will generate its own trajectory based on the spatial position error (e). The overload levels (*O*) in the thrusters determine the UUV’s adaptation to propeller jamming situations.

This network is based on neurobiological principles and combines associative learning and vector associative map learning techniques to generate transformations between the spatial coordinates of the UUV and the velocity coordinates of its thrusters [44]. The design of the SODMN is based on the idea that the nervous systems of animals are capable of generating adaptive behaviors and motor control through self-organization and unsupervised learning [12]. This bioinspired approach has been explored in several similar control algorithms, such as the Vector Integration To Endpoint (VITE) neural network model [45], which uses a vector integration mechanism to generate smooth and controlled trajectories in the motor control of robots. The control architecture of the SODMN also bears similarities to cerebellar-based adaptive motor control models [29,46]. These models use neural networks to learn and adapt the sensorimotor transformations necessary for the precise control of movements, similar to how the SODMN learns to map spatial directions to thruster speed commands.

Furthermore, the ability of the SODMN to adapt to thruster failures and maintain the stable control of the UUV is comparable to neural-network-based fault-tolerant control approaches [19,47]. These approaches use the learning and adaptation capacity of neural networks to compensate for failures in actuators or sensors and maintain an acceptable performance of the control system.

### 3.3. SODMN Description

**Figure 3:**
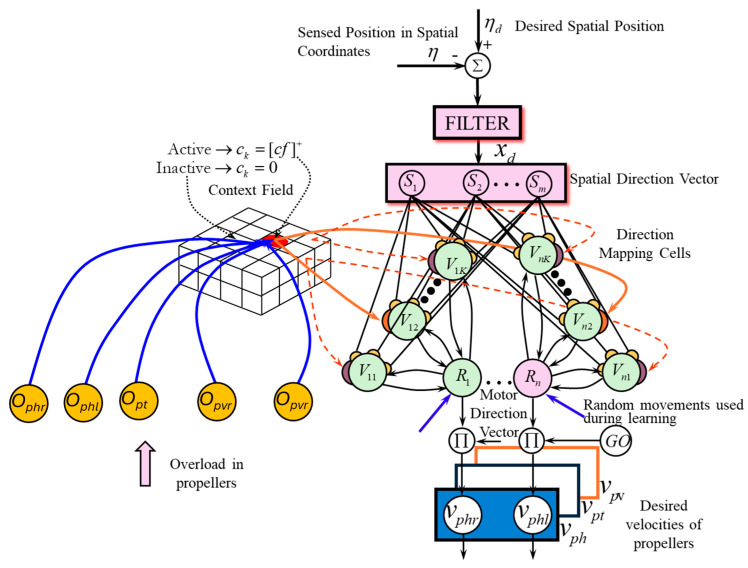
SODMN with context fields activated by overload in the thrusters.

The Self-Organizing Direction Mapping Network (SODMN) is a complex neural network architecture that consists of several interconnected components, as shown in Figure 3. These components work together to enable the system to learn and adapt to various navigational contexts and situations.

At the heart of the SODMN lies the context map, which is responsible for selecting the appropriate elements of the direction mapping network based on the current angular velocities of the UUV’s thrusters. This map contains a set of cells that can be activated or deactivated depending on the specific range of thruster speeds. By doing so, the context map allows the SODMN to adapt its neural connections and weights to suit the current navigational context.

The spatial direction vector (DVs) is another crucial component of the SODMN. It represents the desired direction of movement for the UUV in three-dimensional space. The DVs consists of a set of cells, each corresponding to a specific spatial direction. The activity levels of these cells ($S_{1}$, $S_{2}$, ⋯, $S_{m}$) indicate the importance or priority of each direction in the overall desired motion of the vehicle.

The DVs cell activities, $S\in R^{m}$, are driven by the desired spatial direction, $x_{d}\in R^{m}$, computed from the difference between the desired spatial position and the current spatial position of the robot. The DVs cell activities are given as

(2) $$\frac{d}{dt}S_{j}=\delta \left[x_{dj}-S_{j}\right],$$

where δ represents a gain factor that regulates the rate of integration speed.

The direction mapping network is a collection of neural networks associated with the context map. These networks are responsible for transforming the spatial directions encoded in the DVs into appropriate motor commands for the UUV’s thrusters. Each direction mapping cell $V_{nk}$ receives inputs from all the cells in the DVs, but only connects to a single $R_{n}$ cell in the motor direction vector (DVm). During the learning process, the weights of these connections are adjusted based on the difference between the desired spatial directions and the actual velocities of the thrusters. Direction mapping cells with activity $V_{nk}$ compute the difference between the weighted DVs input and the DVm activity. The activities of the $V_{nk}$ cells are defined as

(3) $$\frac{d}{dt}V_{ik}=\alpha \left[-V_{ik}+c_{k}\left(\sum_{j}z_{jik}S_{j}-R_{i}\right)\right],$$

where α is a time constant, the coefficients $c_{k}$ (*k* = 1, ⋯, *K*) represent inhibition from the context field, and $z_{jik}$ represents the element of the inverse mapping, which multiplies the *j*th spatial components to contribute to the *i*th velocity component. Here, the spatial representation contains *m*-components and *n* is the number of independent actuators.

The motor direction vector (DVm) represents the desired speeds for each of the UUV’s thrusters. Its dimensionality is equal to the number of independent thrusters on the vehicle. The activities of the cells in the DVm are used to generate the actual speed commands sent to the thrusters, allowing the UUV to move in the desired direction.

The motor direction cell activities, $R\in R^{n}$, are driven by the $V_{nk}$ during performance and by actuator velocities $V_{p}$ during learning, and are given as

(4) $$\frac{d}{dt}R_{i}=\delta_{R}\left[\left(1-e\right)\left(\sum_{j}V_{ik}-R_{i}\right)+e\left(V_{pi}-R_{i}\right)\right],$$

where $\delta_{R}$ is a gain that controls the integration speed rate and e is the activation of an endogenous random generator (ERG). In the learning phase, the ERG circuit is activated, e = 1, and the $R_{i}$ cells are driven to sense velocities on the actuators, $V_{pi}$. During the performance, the ERG circuit is inactive, e = 0, and the input is the sum of the $V_{nk}$, only one of which will actively process inputs.

The Motor Present Direction Vector (PDVm) cell activities ($V_{p}^{d}$) are given by

(5) $$\frac{d}{dt}V_{pi}^{d}=\alpha_{v}\left[R_{i}\cdot g+e\cdot V_{pi\;ERG}\right],$$

where $\alpha_{v}$ is a gain that controls the integration speed rate. In the model, the velocities’ commands of the actuators are represented by $V_{pi\;ERG}$, and are given by ERG in the learning phase.

Learning occurs by reducing weights in line with the product of the presynaptic and postsynaptic activities. Hence, the learning rule is derived using the gradient descent algorithm. Training is conducted through the generation of random movements, and the resultant angular velocities along with the observed spatial velocities of the biomimetic robot are used as training vectors for the direction-mapping network. The network’s weights are determined using a version of the gradient descent algorithm adapted for discrete implementation as

(6) $$z_{jik}\left(t+1\right)=z_{jik}\left(t\right)+\beta \left(V_{pi}-\sum_{j}z_{jik}x_{dj}\right)x_{dj},$$

where β is the learning rate and is a positive constant gain.

During the learning phase in a specific context, labeled as the kth, where e equals 1 (indicating the ERG circuit is activated), it is observed that:

$$S_{j}\;\to x_{dj},\;R_{i}\;\to \;V_{pi},\;V_{pi}^{d}=V_{pi}\;\to \;V_{piERG},\;\mathrm{and}\;V_{ik}\;\to \;\left(\sum_{j}z_{jik}S_{j}-R_{i}\right)$$

In the SODMN model, operations are learned autonomously through repetitive action–perception cycles that involve recoding proprioceptive information related to the underwater robot.

### 3.4. Resilience and Adaptability of SODMN to Thruster Failures

Unmanned underwater vehicles equipped with the Self-Organizing Direction Mapping Network (SODMN) neural network demonstrate a remarkable ability to adapt and maintain stable and effective control, even in the face of propeller loss due to jamming. This resilience is due to the SODMN’s ability to learn and modify its connections and synaptic weights, allowing the UUV to continue navigating and performing its tasks, despite the loss of a critical thruster.

The adaptation process begins with the detection of thruster loss, which is accomplished through continuous monitoring of the UUV’s sensors, such as current sensors or encoders in the thrusters. When an anomaly is detected, such as excessive current or a lack of rotation in a thruster, the UUV control system alerts the SODMN of the loss.

Once the thruster loss is detected, the SODMN selects an appropriate contextual map to better adapt to the new situation. This new map assigns greater importance to cells that represent velocity configurations that do not depend on the lost thruster. This allows the SODMN to adapt its behavior and generate velocity commands for the remaining thrusters, thus compensating for the loss of the damaged thruster.

The direction map cells, responsible for performing the transformation between the UUV’s spatial coordinates and the velocity coordinates of the thrusters, also adjust their synaptic weights to adapt to the new situation. The SODMN utilizes a learning mechanism based on the modified gradient descent algorithm to adjust the weights of the connections between the direction map cells and the motor direction vector (DVm) cells. During this adaptation process, the direction map cells corresponding to the lost thruster gradually reduce their influence on the DVm, while the cells associated with the remaining thrusters increase their contribution to compensate for the loss.

Furthermore, the GO signal, which acts as a non-specific multiplicative gate and controls the overall speed of the UUV’s movement, can also be adjusted in response to the loss of a thruster. In situations where the UUV’s propulsion capability is compromised, the GO signal can be modulated to reduce the overall speed of movement and ensure the safe and stable control of the vehicle.

### 3.5. Navigation over Caulerpa Prolifera Meadows

The Mar Menor, a saline coastal lagoon located in the region of Murcia, southeastern Spain, is characterized by its shallow waters and great biodiversity. However, the invasive alga Caulerpa Prolifera, which entered the lagoon in the 1970s due to the widening of the channel connecting the Mar Menor with the Mediterranean Sea, now covers a significant portion of its seabed. For continuous observation plans and scientific analyses of the Mar Menor, which aim to monitor and improve the knowledge about the environmental status of the lagoon and its watershed through continuous observation, scientific analyses, and numerical models, it is important to document the Caulerpa Prolifera meadows through photography, video, and sonar. The results of these studies guide decision making and future actions for the conservation and recovery of this ecosystem.

To carry out a detailed exploration of the Caulerpa meadows, parallel transects are designed with lengths and distances between them adapted for each mission. This methodology allows for a comprehensive study of the area, ensuring a systematic and complete collection of data on the distribution and density of these algae. At the beginning of these observation missions, the operational depth of the UUV and the optimal distance to the algae are precisely determined to ensure the capture of high-quality images and videos. Due to the turbidity of the water, it is necessary to maintain considerable proximity between the UUV and the algae, navigating just above them and at a low speed. However, this proximity increases the risk of larger algae reaching and obstructing the submarine’s propellers, particularly the steering propeller located at the bottom, which often leads to the entanglement of algae in the propellers, especially in the rudder, causing them to become blocked. In the Section 4, a clear example of this complication is illustrated, showing the propeller jamming in a UUV, leaving the vehicle completely inoperative and requiring its immediate recovery from the water for repair and restoration.

As the algae accumulate on the propellers, the load on the motors progressively increases until reaching a point of total blockage, which carries a significant risk of mechanical or electronic breakdown. To mitigate this risk, the control system measures the overload of each of the thrusters through the current intensity. When the overload level exceeds a limit, early warnings are issued, which are used to interrupt the mission, extract the submarine from the water, and perform the necessary cleaning of the propellers. The proposed control system tries to reduce these types of maneuvers, as they not only interrupt the continuity of the mission, but also involve a considerable loss of resources and time to resume operations from the point of interruption. Conventional control systems struggle to operate UUVs with blocked thrusters. If there is a disconnection of any motor, the vehicle must stop and be taken out of the water to restore the thrusters by cleaning them of entangled algae. However, with the new bioinspired control system, the detection of motor overloads triggers a reconfiguration of the control system that allows for operation even with blocked thrusters. This adaptive feature enables the UUV to continue its mission without interruption, optimizing resource utilization and minimizing downtime. The bioinspired control system’s ability to adapt and maintain functionality in the presence of motor failures represents a significant advancement in the field of underwater robotics, enhancing the efficiency and effectiveness of scientific exploration and monitoring missions in challenging environments such as Caulerpa Prolifera meadows.

The mission execution process consists of several stages, including configuration, planning, learning, and the exploration mission. During the configuration stage, the UUV, comprising both the underwater vehicle and the surface buoy, is deployed in the marine environment. Various parameters, such as the mission speed and settings for the vision and sonar systems, are adjusted. One crucial configuration is determining the optimal distance between the UUV and the seabed for the mission. This distance is experimentally established by finding the ideal range at which the algae can be clearly observed by video and photographic cameras, while also adjusting the lighting conditions using the submarine’s spotlights. Factors such as water turbidity, natural lighting conditions at depth, and artificial lighting from spotlights influence the determination of this distance. The selected distance represents the separation that the underwater robot will maintain from the algae throughout the entire exploration mission. If the distance to the algae is too small, there is a risk of the algae becoming entangled in the propellers, particularly the bottom-mounted rudder propeller.

The planning stage involves generating routes and transects for the mission. Transects are conducted over areas with consistent depth levels to ensure lighting conditions similar to those established during the configuration stage. Mission planning software, along with data from previous bathymetric surveys or underwater mapping, is utilized to create the plan. Key parameters such as the starting point, ending point, length of the lanes, and separation between them are defined during this stage.

The learning phase involves generating context fields and precisely adjusting the neural weights in the neural networks. The learning algorithms generate random movement commands for the thrusters, and the resulting speeds along the *X*, *Y*, and *Z* axes are measured. These results are used to perform input–output correlation by adjusting the weights of the neurons. The learning process with blocked thrusters is conducted by disconnecting either the rudder motor or one of the propulsion thrusters, as the vehicle is capable of maintaining its operation without these motors. This one-minus thruster learning allows for the motion and propulsion correlations for these specific thruster failure scenarios to be learned.

The learning mission represents the initial attempt to complete the entire mission, although it is still subject to ongoing learning processes. During this stage, currents in the electric motors, particularly the rudder motor, are monitored. If an increase in power is detected due to the entanglement of algae in the propeller, the robot halts its movement and initiates a learning process to acquire new correlations and generate new context fields specific to the situation of propellers with entangled algae.

Once the underwater robot has successfully completed the preceding stages, it is prepared to undertake exploration missions. At this point, the neural algorithms are fully trained and the UUV can execute the mission under normal operating conditions.

## 4. Results

Experiments were conducted to validate the bioinspired control architecture in the Mar Menor. The main objective of these experiments was to demonstrate that the UUV exhibits resilient behavior to propeller failures caused by the entanglement of algae in the propellers and can increase the distance traveled by the UUV over the Caulerpa Prolifera meadows while reducing interruptions caused by the entanglement of algae in the propellers. This challenge is particularly relevant in environments with dense underwater vegetation, where the risk of propeller obstruction is high and can compromise the continuity of the mission and the integrity of the vehicle.

By validating the ability of the bioinspired control architecture to adapt and maintain the stable control of the UUV in the presence of propeller failures, these experiments seek to demonstrate the effectiveness of this innovative approach to improve the robustness and reliability of exploration and monitoring missions in challenging underwater environments.

### 4.1. Learning Phase

The learning phase aims to adjust the weights of the neurons in the context fields *V_nk_*. These context fields are determined for different situations of overload and blockage in the thrusters, caused by the entanglement of algae in the propellers. During this phase, sequences of random movements are generated on the motor direction vector (DVm), which are applied to the UUV’s thrusters. Next, the results of the vehicle’s spatial movement are measured in the spatial direction vector (DVs). The weights of the neurons are adjusted using Equation (6).

In the first stage of learning, the system adjusts the neural weights of the operating contexts defined in Table 1. This table includes all possible cases in which the UUV can develop its full mobility, albeit with different combinations of blocked thrusters. In this table, the thrusters can be active or blocked, and in the case of being active, they do not present any overload. To simulate the blockage of the thrusters due to algae entanglement, a safety blocking command is activated on the corresponding thruster. This command renders the thruster inactive, preventing it from responding to the movement commands applied to it. In this way, a scenario in which the motor is blocked by an obstruction caused by algae is effectively reproduced. This approach allows the system to learn and adapt to different thruster configurations, improving its ability to maintain the mobility and control of the UUV in the presence of thruster failures.

**Figure 4:**
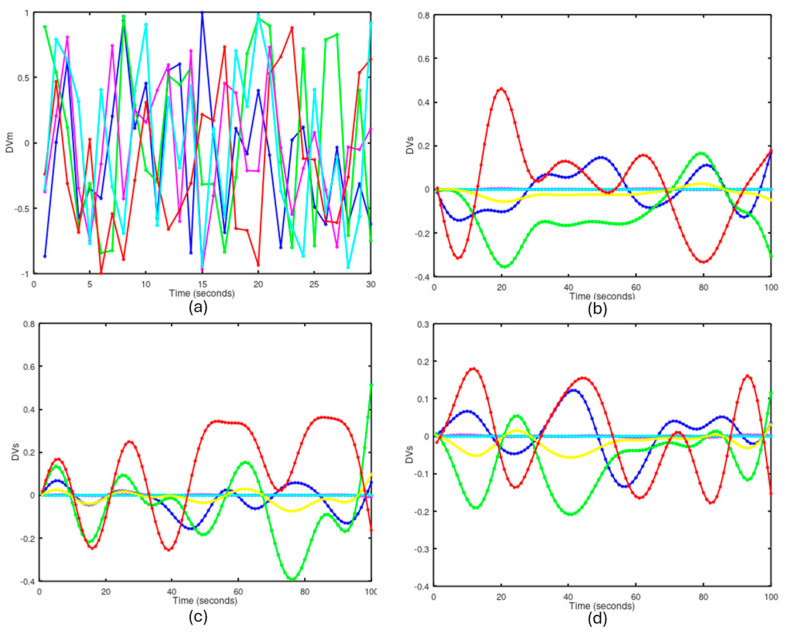
DVm and DVs vectors during the learning processes. (**a**) DVm values generated by the ERG. (**b**) DVs values in learning with 5 thrusters. (**c**) DVs with the right horizontal thruster blocked. (**d**) DVs with the right vertical and horizontal thrusters blocked.

Figure 4 shows the variables DVm and DVs in the learning processes for some context fields. Figure 4a depicts the DVm values applied to the thrusters in the learning process of context 1 (blue for horizontal left, green for horizontal right, magenta for rudder, red for vertical left, and cyan for vertical right). Each of these values was applied for one second and the process lasted 20 min, during which, 1200 different movements were randomly generated. This graph (a) shows the random and normalized DVm values corresponding to the first 30 s of the learning process. Graph (b) shows the values of the DVs vector in the learning process of context 1, which represents the normalized linear and angular velocity values in *XYZ*. The values DVs1, DVs3, and DVs6 (blue for frontal direction, green for altitude, and red for yaw rotation) are the most sensitive to the learning process. In contrast, the values DVs2 (transverse velocity in *Y*, magenta) and DVs4 (rotation velocities in *X*) are negligible, as the UUV has no mobility in those directions. Graph (b) shows the first 100 s of the DVs variables. For these tests, the vehicle was positioned at a depth of 5 m in calm sea conditions without currents. Graph (c) shows the DVs values in the learning of context 2, with the right horizontal thruster blocked, and graph (d) shows the DVs values in context 7, with both the right horizontal and right vertical thrusters blocked.

In the second phase of learning, the UUV is operated in the algae meadows, and learning is carried out in new contexts with various levels of overload caused by different degrees of algae entanglement in the thrusters. A 100% overload level causes complete motor blockage. The criterion followed for the generation of new contexts was that, when an increase of approximately 20% in the percentage of propeller overload is reached compared to the previous values, the mission is stopped, and the UUV performs a learning process under those conditions, following the same procedure as that in the previous phase. This operation was performed repeatedly as the propellers became obstructed with algae, which was conducted at four different points along the UUV’s route. At each of these new points, new context fields were created to adapt to the specific overload conditions. Table 2 shows the new contexts generated for the indicated overload levels, allowing the system to learn and adapt to situations of progressive algae entanglement in the thrusters during the UUV’s operation in the marine meadows.

### 4.2. Thruster Failure Experiment

**Figure 5:**
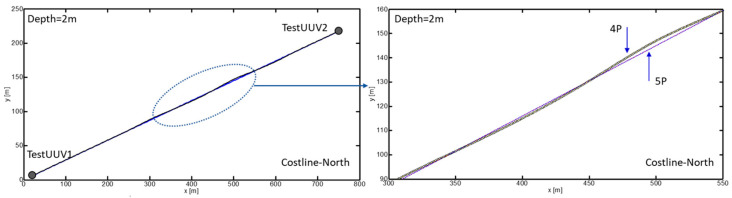
UUV navigation trajectory with one propeller failure when reaching a TestUUV2 target (750, 225, 2) m.

After completing the learning phase, the control system was tested by inducing a failure in the rudder propeller to evaluate its responsiveness to anomalies. The left graph of Figure 5 illustrates the route followed by the UUV when experiencing this failure while heading towards the target destination. The starting point, called TestUUV1, is located at coordinates (20, 10, 2) m, while the destination point, TestUUV2, is situated at coordinates (750, 225, 2) m. To ensure accuracy in distance measurement, an acoustic positioning system was employed.

Initially, a navigation route was established for the UUV using its five thrusters, operating in context field 1. Subsequently, during the vehicle’s movement, a failure in the rudder propeller was provoked by issuing a blocking command to this motor. This simulated incident was activated when the UUV had advanced to the position of (180, 53, 2) m from its starting point. Faced with this eventuality, the bioinspired control algorithm demonstrated its adaptive effectiveness by automatically switching from context 1 to context 6, allowing the UUV to continue its movement towards the target using only four propellers. The route undertaken in this induced failure condition is detailed in the right graph of Figure 5.

**Figure 6:**
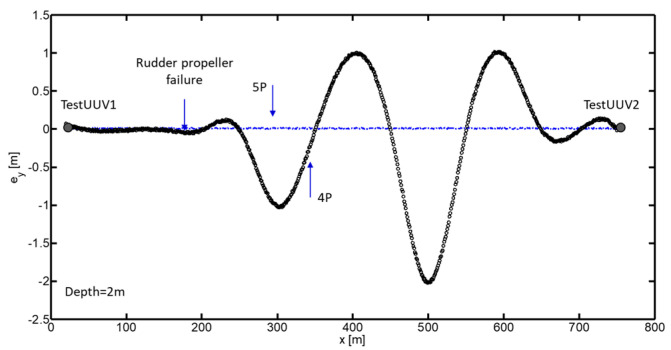
Deviation error with respect to the generated trajectory when 5 thrusters are used.

Figure 6 shows the deviation error with respect to the generated trajectory when using five thrusters (5T configuration) and when a failure in the rudder thruster is provoked. In the 5T configuration, the generated trajectory is represented by a solid line, indicating that the UUV follows the planned route precisely when all thrusters are functioning correctly. However, when the rudder thruster is blocked, the trajectory deviates from the planned route, which is represented by a dashed line. The maximum deviation error observed in the configuration with four thrusters (4T) is approximately 2 m compared to the originally planned trajectory with five thrusters. This relatively small error highlights the effectiveness of the bioinspired control algorithm in compensating for the thruster failure and maintaining the stable control of the UUV. Despite the deviation caused by the rudder thruster failure, the UUV is capable of continuing its navigation towards the destination point TestUUV2 using the remaining four thrusters. This demonstrates the ability of the bioinspired control system to adapt and maintain the vehicle’s course, even in the presence of thruster failures.

### 4.3. Comparison of Results with Conventional Control

**Figure 7:**
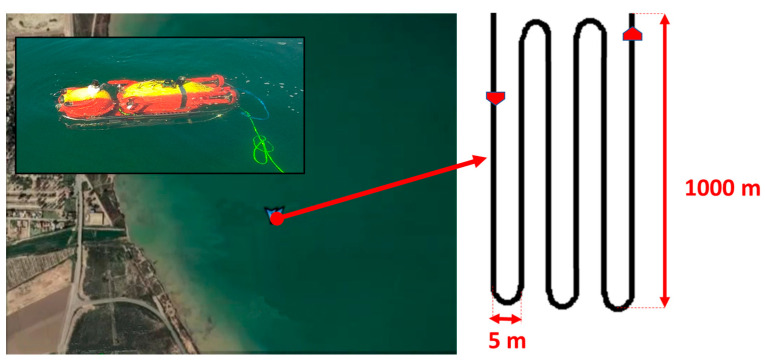
Mission planning in underwater vegetation meadows.

To carry out a detailed exploration of the Caulerpa meadows, transects with a length of 1000 m were designed, maintaining a distance of 5 m between each of them, as illustrated in Figure 7. This methodology allows for a comprehensive study of the area, ensuring a systematic and complete collection of data on the distribution and density of these algae.

**Figure 8:**
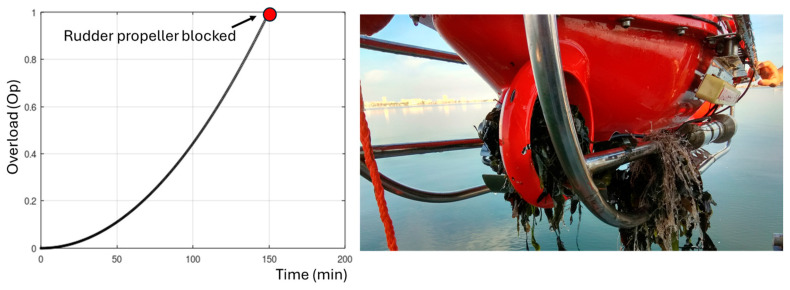
Mission cancellation with conventional control due to rudder propeller jamming.

An experiment was conducted with conventional control to complete the route shown in Figure 7. In this case, the orientation is controlled by the rudder thruster, while forward and backward movement is controlled by the horizontal thrusters and immersion is controlled by the vertical thrusters. The vehicle was programmed to follow the submerged fields of Caulerpa Prolifera, maintaining a distance of 0.5 m from the seabed. The mission had to be stopped at 4.8 km due to the jamming of the rudder propeller, as the vehicle lost its ability to control its orientation. The left graph in Figure 8 shows the evolution of the normalized overload signal in the rudder, which reaches the overload limit level at minute 148. The photograph included in Figure 8 shows how the rudder propeller was completely jammed and inoperative.

**Figure 9:**
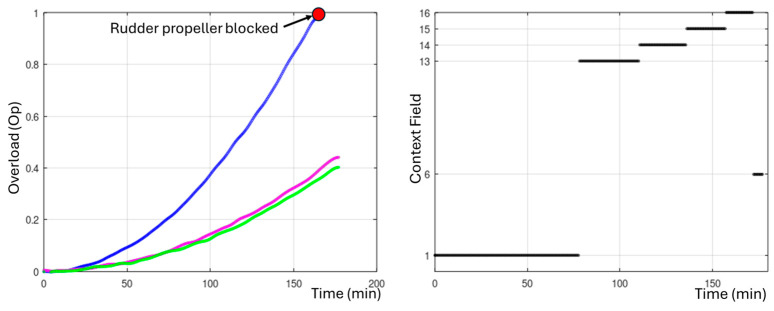
Successful mission completion with a bioinspired controller, despite the propeller jamming.

Subsequently, the same route was performed using the bioinspired control system. Figure 9 shows the switching of context fields until the mission is completed. Initially, the UUV operates with context field 1. At 77 min, the controller detects an overload above 0.2 (20%) in the rudder thruster and switches to context field 13. When the overload exceeds 0.4 (40%) at 110 min, it switches to context field 14. With an overload of 0.6 (60%) at 135 min, it switches to context field 15. With an overload of 0.8 (80%) at 156 min, it switches to context field 16. Finally, when the overload exceeds 0.95 (95%) at 171 min, the motor is blocked, and context field 6 is activated, corresponding to the case of a blocked motor.

**Figure 10:**
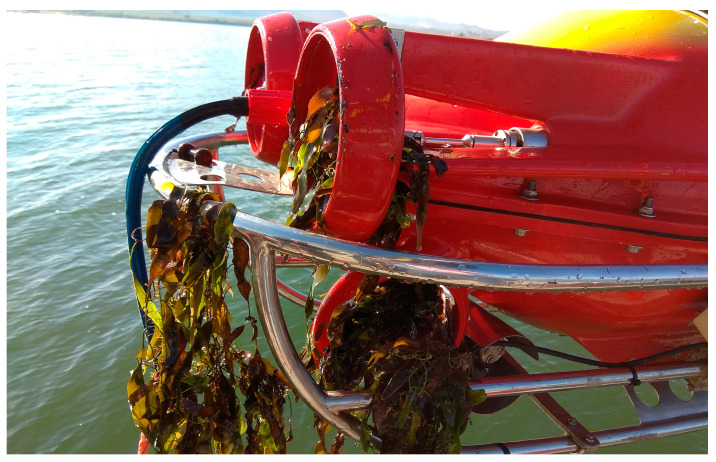
Propellers after completing the exploration mission.

Ultimately, the UUV managed to complete the mission. Figure 10 shows an image of the state of the thrusters. The rudder thruster was completely blocked due to entanglement with algae, however, the horizontal thrusters were not blocked and were able to complete the mission.

The implemented algorithm plays a crucial role in finding an alternative strategy that allows the UUV to continue operating by adjusting the propulsion propellers. The selected context fields lead to a situation in which the orientation control produced directly by the rudder thruster in conventional control is replaced by the power differential between the port and starboard thrusters, an ingenious solution that compensates for the loss of directional control provided by the rudder propeller. Since these propulsion propellers are located further away from algae concentrations, they experience less entanglement, ensuring continuous and resilient operability until the end of the mission. Figure 10 illustrates the state of the thrusters after completing the exploration, highlighting their ability to remain operational without the need for interruptions and mission cancellation due to algae cleaning from the propellers or breakdowns.

### 4.4. Results of the Exploration of Caulerpa Prolifera Fields

The mission was successfully completed, achieving a comprehensive documentation of 4 km through transects of 1 km in length and separated by 5 m from each other, following the route shown in Figure 7. The UUV demonstrated a remarkable capacity for adaptation to unforeseen events, adjusting its operation in response to specific complications, such as the progressive obstruction of the rudder propeller until its final blockage. This demonstrates the operational flexibility of the vehicle, which, even with the rudder propeller immobilized, continues to operate effectively.

**Figure 11:**
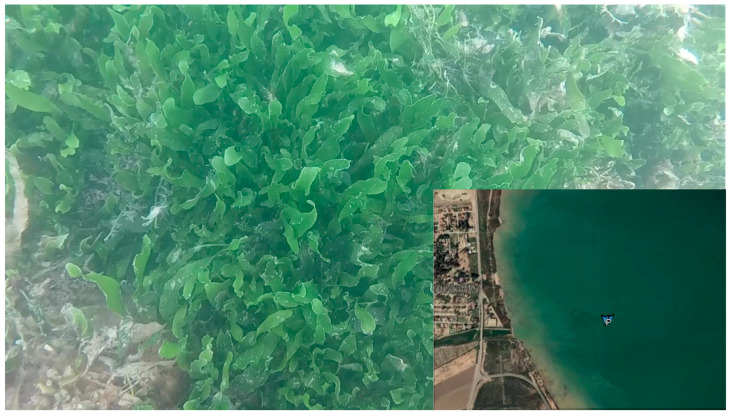
Photographs taken by UUV.

During the exploration mission, videos, photographs, and sonar records of the seabed were captured for further analysis. Figure 11 presents a detailed image of the Caulerpa Prolifera meadows captured along the exploration transects, providing a clear view of the underwater ecosystem under study.

**Figure 12:**
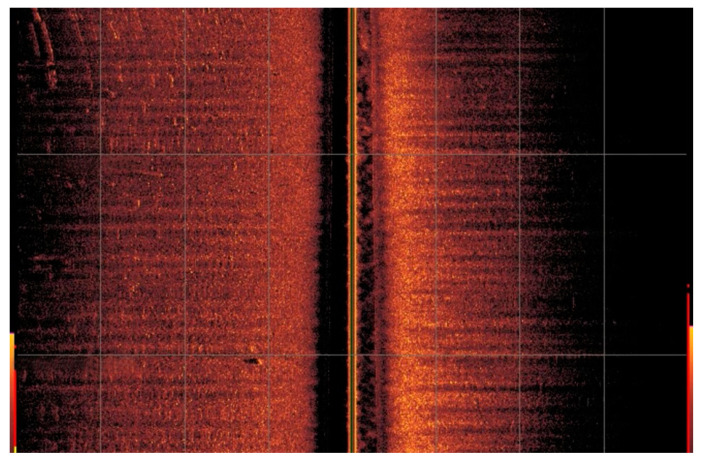
UUV side-scan sonar images.

Figure 12 shows an image obtained by side-scan sonar of the area in question, revealing a predominantly flat terrain with the presence of small rocky objects. This sonar visualization provides a detailed perspective of the underwater topography, highlighting the physical characteristics of the seafloor.

## 5. Discussion

The results obtained in this study demonstrate the effectiveness of the bioinspired control architecture based on the Self-Organizing Direction Mapping Network (SODMN) neural network in providing a UUV with adaptation and resilience capabilities in the face of thruster failures during exploration and monitoring missions of the submerged Caulerpa Prolifera meadows in the Mar Menor.

The ability of the UUV to continue operating effectively, even in situations of algae entanglement in the propellers, thanks to the adaptability of the SODMN neural network, represents a significant advance compared to conventional control systems. As illustrated in Figure 8, UUVs using traditional control algorithms often require mission interruption and vehicle recovery for repair when a thruster blockage occurs. In contrast, the bioinspired control architecture allows the UUV to adjust its neural weights and connections to compensate for the loss of a thruster and generate appropriate velocity commands for the remaining thrusters, thus maintaining stable and effective control (Figure 5 and Figure 6).

The experimental design involves several crucial phases, including tuning, planning, learning, learning mission, and the exploration mission. Each of these phases plays a fundamental role in evaluating and validating the performance of the bioinspired control system.

During the tuning phase, critical adjustments were made to the UUV’s operational parameters, such as the mission speed and configuration of the vision and sonar systems. A key aspect of this phase was determining the optimal distance between the UUV and the algae to ensure the capture of high-quality images and videos.

This distance was established experimentally, taking into account factors such as the water turbidity, lighting conditions, and need to avoid algae entanglement in the propellers. The proper determination of this distance is essential for mission success, as a balance between the quality of the collected data and vehicle safety is critical.

In the planning phase, routes and transects were generated to exhaustively cover the study area. The careful selection of start and end points, as well as the length and separation of the transects, demonstrates a methodical approach to ensuring systematic and comprehensive data collection. This detailed planning lays the foundation for an efficient and effective exploration of the Caulerpa Prolifera meadows.

The learning phase is a crucial component of the experiments, as it allows the bioinspired control system to adapt and learn from interactions with the environment. During this phase, the UUV generates random motion commands to the thrusters and measures the resulting velocities along each axis. This process enables the control algorithm to establish correlations between the inputs and outputs, adjusting the neuron weights accordingly. What distinguishes this approach is the inclusion of simulated failure scenarios, such as disconnecting a motor or obstructing the propellers with algae. By exposing the system to these adverse situations during learning, its ability to adapt and maintain control under real failure or performance degradation conditions is enhanced.

The learning mission represents the first attempt to fulfill the objectives of the complete mission while subjecting the system to a learning and adaptation process under the conditions of partial thruster overload. During this phase, the currents in the electric motors are closely monitored to detect possible power increases due to the entanglement of algae in the propellers. If such a situation is detected, the UUV halts its movement and initiates a learning process to adapt to the new condition. This approach demonstrates the system’s ability to respond in real time to encountered challenges and adjust its behavior accordingly.

The exploration mission puts the performance of the UUV and its bioinspired control system to the test in real operating conditions. During this phase, the vehicle navigates through the planned transects, capturing videos, photographs, and sonar records of the Caulerpa Prolifera meadows. The ability of the UUV to successfully complete the mission, despite the challenges encountered, such as algae entanglement in the propellers, demonstrates the robustness and adaptability of the control system.

A notable aspect of the experiments is the simulation of propeller failures to evaluate the responsiveness of the bioinspired control algorithm. The scenario in which a disconnection failure is provoked in the rudder thruster during navigation provides a critical test of the system’s resilience. The results show that the UUV is capable of continuing its displacement towards the objective using only four propellers, thanks to the adaptive reconfiguration of the control system.

This finding supports the hypothesis that the bioinspired control architecture can provide the UUV with enhanced resilience against failures and maintain an acceptable performance under adverse conditions.

Furthermore, the control algorithm’s ability to find alternative propulsion strategies, such as generating a power differential between the port and starboard propellers to compensate for the loss of directional control, demonstrates its flexibility and problem-solving capability. This adaptability is especially relevant in challenging environments like the Caulerpa Prolifera meadows, where algae entanglement in the rudder propeller is a constant risk.

The experimental results also highlight the importance of redundancy in UUV propulsion systems. The five-thruster configuration of the vehicle used in this study allows for greater fault tolerance and three-dimensional maneuverability, even when two of the thrusters are inoperative.

This redundancy, combined with the bioinspired control system, significantly improves the resilience and reliability of the UUV in submerged aquatic vegetation monitoring missions.

These findings are consistent with previous studies that have explored the application of bioinspired and biomimetic algorithms in the field of robotic resilience [48,49,50,51]. These approaches are based on emulating the principles and mechanisms of adaptation, learning, and robustness present in biological systems, with the aim of creating more resilient and autonomous robotic systems [49]. The SODMN neural network’s ability to adapt to thruster failures and maintain the stable control of the UUV is comparable to neural-network-based fault-tolerant control approaches [25,52], which leverage the learning and adaptation capabilities of neural networks to compensate for actuator or sensor failures and maintain an acceptable control system performance.

In summary, the experiments conducted in this study provide strong validation of the effectiveness and adaptability of the proposed bioinspired control architecture for UUVs in submerged aquatic vegetation meadow monitoring missions. The experimental design encompasses multiple phases, from tuning and planning to learning and exploration, allowing for a comprehensive evaluation of the system’s performance under real and simulated operating conditions. The obtained results support the hypothesis that bioinspired approaches can significantly enhance the robustness, adaptability, and resilience of control systems in autonomous vehicles, especially in challenging and dynamic environments such as the Caulerpa Prolifera meadows in the Mar Menor. These findings have important implications for the future development of UUVs and bioinspired control systems and lay the foundation for further research in this field.

## 6. Conclusions

In this study, a bioinspired control architecture for the autonomous, adaptive, and resilient navigation of a UUV on missions to monitor submerged aquatic vegetation meadows has been presented. The proposed architecture is based on a self-organizing directional mapping network (SODMN) that combines associative learning with vector associative map learning to generate transformations between the UUV’s spatial coordinates and the velocity coordinates of its thrusters.

The experimental results obtained demonstrate the effectiveness and adaptability of the bioinspired control architecture in a real operating environment, specifically in the Caulerpa Prolifera meadows in the Mar Menor. The UUV, equipped with the proposed control system, successfully completed exploration missions, capturing valuable data on the distribution and density of algae. It navigated challenging conditions, even with its propellers entangled in algae.

One of the main strengths of the bioinspired control architecture is its ability to adapt and reconfigure the UUV’s propulsion scheme in response to adverse situations, such as algae entanglement in the propellers or the loss of functionality of a thruster.

The experiments conducted, including the simulation of failures in the rudder propeller, demonstrated the robustness and resilience of the control system, allowing the UUV to continue its mission using redundant thrusters and alternative propulsion strategies.

These results have significant implications for the future development of autonomous vehicles and bioinspired control systems in a wide range of applications, from the exploration and monitoring of marine ecosystems to the inspection and maintenance of underwater infrastructures. The ability of bioinspired approaches to endow robotic systems with adaptation and resilience skills similar to those observed in biological systems opens up new possibilities for the design of more efficient and robust robots.

Another area of interest for future research is the optimization of learning algorithms and the reduction in the time required for the control system to adapt to new situations. The incorporation of advanced techniques, such as reinforcement learning and deep learning, could accelerate the adaptation process and improve the efficiency of the control system.

In conclusion, this study demonstrates the potential of the bioinspired control architecture to enhance the adaptability, resilience, and performance of UUVs in monitoring missions of vulnerable marine ecosystems. The results obtained highlight the importance of bioinspired approaches in the design of advanced robotic systems and provide new perspectives for the development of more efficient and robust control algorithms. The integration of biological principles and the emulation of adaptation strategies observed in nature have the potential to revolutionize the field of autonomous robotics and significantly contribute to the conservation and sustainable management of marine ecosystems.

As we move forward in this direction, it is essential to foster interdisciplinary collaboration among experts in robotics, biology, ecology, and other relevant disciplines to address the complex challenges facing our oceans and develop innovative and sustainable solutions. The combination of bioinspired approaches and advanced technologies, such as in UUVs, offers promising opportunities to enhance our understanding of marine ecosystems, monitor the impacts of climate change and human activities, and support efforts towards the conservation and sustainable management of these valuable natural resources.

## Figures and Tables

**Table 1:** Learning contexts for situations with blocked thrusters.

Context	Horizontal Right	Horizontal Left	Vertical Right	Vertical Left	Rudder
1	Active	Active	Active	Active	Active
2	Blocked	Active	Active	Active	Active
3	Active	Blocked	Active	Active	Active
4	Active	Active	Blocked	Active	Active
5	Active	Active	Active	Blocked	Active
6	Active	Active	Active	Active	Blocked
7	Blocked	Active	Blocked	Active	Active
8	Blocked	Active	Active	Blocked	Active
9	Active	Blocked	Blocked	Active	Active
10	Active	Blocked	Active	Blocked	Active
11	Active	Active	Blocked	Active	Blocked
12	Active	Active	Active	Blocked	Blocked

**Table 2:** Learning contexts for thruster overload situations.

Context	Horizontal Right	Horizontal Left	Vertical Right	Vertical Lelf	Rudder
13	0%	0%	0%	0%	21.3%
14	11.3%	10.4%	0%	0%	42.5%
15	14.6%	13.5%	0%	0%	62.1%
16	17.2%	16.7%	0%	0%	81.7%

## Data Availability

Data are contained within the article.
